# ﻿Two new species of *Nitocrella* (Copepoda, Harpacticoida) from a spring in Loei Province, northeastern Thailand

**DOI:** 10.3897/zookeys.1240.151021

**Published:** 2025-06-06

**Authors:** Chaichat Boonyanusith, Anton Brancelj, Laorsri Sanoamuang

**Affiliations:** 1 School of Biology, Faculty of Science and Technology, Nakhon Ratchasima Rajabhat University, Nakhon Ratchasima 30000, Thailand; 2 National Institute of Biology, 1000 Ljubljana, Slovenia; 3 Laboratory of Biodiversity and Environmental Management, International College, Khon Kaen University, Khon Kaen 40002, Thailand; 4 Applied Taxonomic Research Center, Department of Biology, Faculty of Science, Khon Kaen University, Khon Kaen 40002, Thailand

**Keywords:** Ameiridae, biodiversity, groundwater species, karstic springs, *Nitocrellagrandicaudis*, *Nitocrellathailandensis*, saturated karstic aquifer, Southeast Asia

## Abstract

Two new species of the genus *Nitocrella* are described from a karstic spring in Loei Province, northeast Thailand. They are the first representatives of the genus *Nitocrella* Chappuis, 1924, described from Thailand. *Nitocrellagrandicaudis***sp. nov**. and *N.thailandensis***sp. nov**. show the peculiar shapes of the inner element of the male P1 basis, the basis of the mandible, and the proximal endite of the maxilla, differing from their congeners. While *N.grandicaudis***sp. nov.** belongs to the *chappuisi* group according to the absence of a proximal inner seta on the third exopodal segment of the fourth swimming leg, *N.thailandensis***sp. nov.** belongs to the *vasconica* group because the seta is present on the segment. Despite the low degree of differentiation in morphological characters, the shape of the female caudal ramus and the proximal seta of the male fifth segment of the antennule suggest that *N.grandicaudis***sp. nov.** differs significantly from the other new species in its mating behavior. A detailed description and illustrations of two new species are provided, as well as a discussion on the divergence of both new species. Some comments on the basic understanding of the reproductive behavior of Harpacticoida are included.

## ﻿Introduction

The genus *Nitocrella* Chappuis, 1924, belongs to the primarily marine copepod family Ameiridae Boeck, 1865, which currently comprises 50 valid genera (Walter and Boxshall 2024). It was established to accommodate the freshwater species *N.hirta* Chappuis, 1924, from Serbia (Chappuis 1924). At present, 63 valid species have been described within the genus (Walter and Boxshall 2024). Since the genus *Nitocrella* was established, many taxa with different exopod armament and endopod segmentation of the swimming legs were added to it. These additions made the taxonomic boundary of the genus *Nitocrella* blurred (Karanovic 2006). Lang (1965) conducted the first thorough taxonomic revision of the genus, narrowing the taxonomic boundary by establishing the genus *Pseudoleptomesochrella* Lang, 1965, to include *P.halophila* (Noodt, 1952), which increases to five species currently (Walter and Boxshall 2024). He classified species with three-segmented endopods on the second to fourth swimming legs as members of the genus *Pseudoleptomesochrella* and species with two-segmented endopods on the second to fourth swimming legs as *Parapseudoleptomesochra* Lang, 1965. However, the boundary between genera was still unclear, even after revision. Petkovski (1976) removed the species with a one-segmented endopod on the fourth swimming leg to the newly established genus *Stygonitocrella*, while the species with three-segmented endopods on the second and third swimming legs and with a two-segmented endopod on their fourth swimming leg were classified as *Nitocrellopsis*. According to Article 13.3 of the International Code of Zoological Nomenclature (ICZN 1999), Petkovski did not designate the type species of the newly established genera, making the generic names invalid. Galassi et al. (1999) and Reid et al. (2003) described other species from the same genera, designated them as type species, and provided revised diagnoses. This led to the generic names being available with their authorship, i.e., *Nitocrellopsis* Galassi, De Laurentiis & Dole-Olivier, 1999, and *Stygonitocrella* Reid, Hunt & Stanley, 2003. Since then, researchers have re-examined and split existing taxa from the genus *Nitocrella* into additional new genera, such as *Novanitocrella* Karanovic, 2004, *Neonitocrella* Karanovic, 2004, *Reidnitocrella* Karanovic & Hancock, 2009, and *Stygonitocrella* (Reid et al. 2003; Karanovic 2004; Karanovic and Hancock 2009). In summary, researchers have separated members of the original genus *Nitocrella* into at least eight different genera, including the original genus *Nitocrella* itself (Wells 2007; Walter and Boxshall 2024).

Petkovski (1976) distinguished three groups of species within the genus *Nitocrella* based on the number of setae on the third exopodal segment of the fourth swimming leg. The species included in the *hirta* group (1^st^ group) are those with three or four elements on the segment, while those in the *chappuisi* and *vasconica* groups (2^nd^ and 3^rd^ groups) have five and six elements on the segment, respectively.

Representatives of the genus *Nitocrella* exclusively inhabit subterranean freshwater or brackish habitats (Galassi and De Laurentiis 1997). The genus shows a wide geographical distribution range. Representatives were first described from the region surrounding the Mediterranean and Black Sea (i.e., Europe and Central and West Asia), and after that from North America, North and East Asia, and Australia, but there were no records from South America and Southeast Asia (i.e., Thailand and neighboring countries) (Defaye and Dussart 2011).

In this paper, two new species of the genus *Nitocrella* are described from a karstic spring in Loei Province (northeastern Thailand). Both new species coexist with the recently described harpacticoid copepod *Elaphoidellapropecabezasi* Boonyanusith, Brancelj & Sanoamuang, 2024 (Boonyanusith et al. 2024). This contribution provides a detailed description and illustration of both new species.

## ﻿Materials and methods

**Figure 1.**
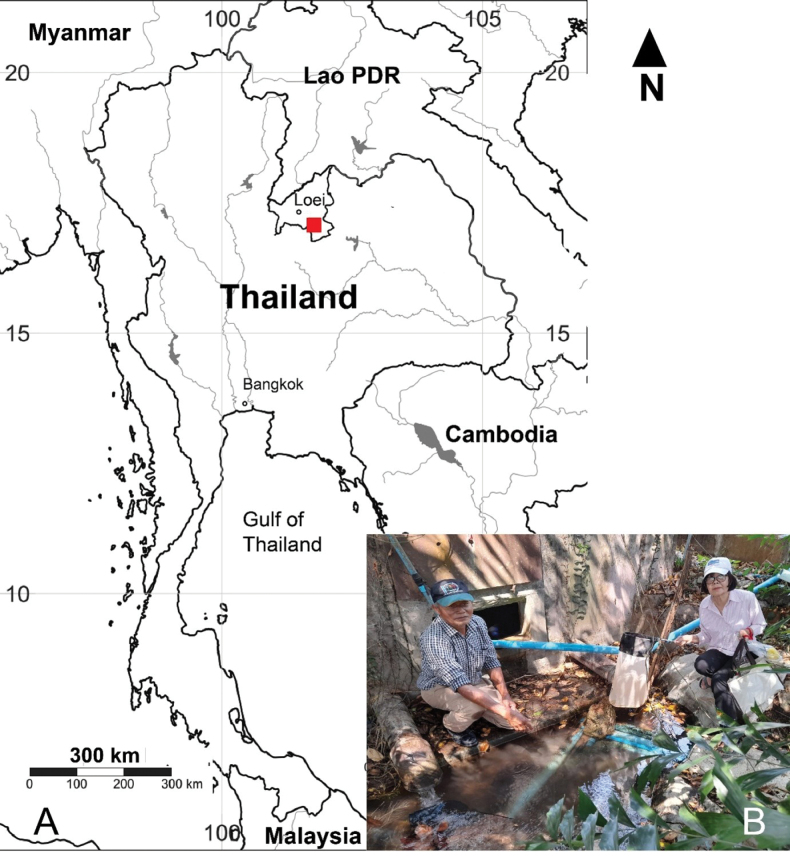
Geographical location and details on sampling site: **A** map of Thailand and location of the sampling site in Loei Province (indicated by red square) **B** entrance to a concrete reservoir from which *Nitocrellagrandicaudis* sp. nov. and *N.thailandensis* sp. nov. were collected.

Copepod samples were collected from a concrete reservoir constructed at a spring mouth, which provides drinking water for the Wat Tham Bodhisattva Temple, Nong Hin District, Loei Province, northeast Thailand (Fig. 1). They were collected by a hand net with a mesh size of 60 µm and put on a spot in ~ 70% ethanol.

Before morphological examination, some specimens were immersed in a mixture of glycerol and 70% ethanol (1:10 v/v) for 30 min, then transferred to a drop of pure glycerol on a glass slide and dissected under a stereomicroscope. Body parts were covered with a coverslip, sealed with nail polish, and examined under a Nikon Eclipse E200 compound microscope at 1000 × magnification. Habitus and appendages were drawn using a drawing tube attached to a compound microscope at 1000 × magnification. The final version of the figures was digitally inked using the CorelDraw 19.0 graphic program.

Abbreviations used in the text are as follows: **ae** = aesthetasc; **Enp** = endopod; **Exp** = exopod; **Enp-1 (2)** = proximal (distal) segment; **Exp-1 (2, 3)** = proximal (middle, distal) segment; **P1–P6** = first to sixth swimming legs. **Seta I–VII** = first to seventh caudal seta; **seta I** = anterolateral accessory seta; **seta II** = anterolateral seta; **seta III** = postereolateral seta; **seta IV** = outer terminal seta; **seta V** = inner terminal seta; **seta VI** = terminal accessory seta; **seta VII** = dorsal seta. For antennule: Roman numerals = segment, Arabic = number of elements. The descriptive terminology follows Huys and Boxshall (1991).

## ﻿Taxonomic section

### ﻿Order Harpacticoida Sars, G.O., 1903

**Family Ameiridae Boeck, 1865**

**Genus *Nitocrella* Chappuis, 1924**

#### ﻿ Nitocrella grandicaudis sp. nov.

Taxon classificationAnimaliaHarpacticoidaAmeiridae

17C5C15D-D6AA-5BEA-A996-7DF8111126B8

https://zoobank.org/DA4E4191-A545-4697-A08D-DE1E88DDEC1C

**Figure 2.**
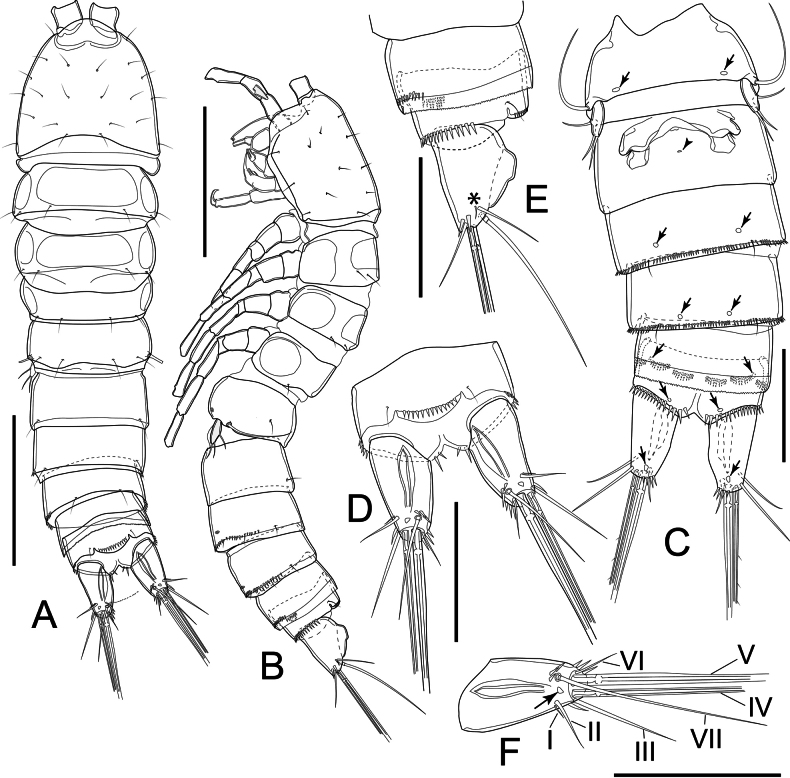
*Nitocrellagrandicaudis* sp. nov., female holotype: **A** habitus, dorsal view **B** habitus, lateral view **C** urosome, ventral view; (arrows and arrowheads indicate integumental pores and copulatory pore, respectively) **D** anal somite and caudal rami, dorsal view **E** anal somite and caudal rami, lateral view (asterisk indicates caudal seta I) **F** left caudal ramus with indication of setation. Arrow indicates integumental pore. Scale bars: 100 μm (**A, B**); 50 μm (**C–F**).

**Figure 3.**
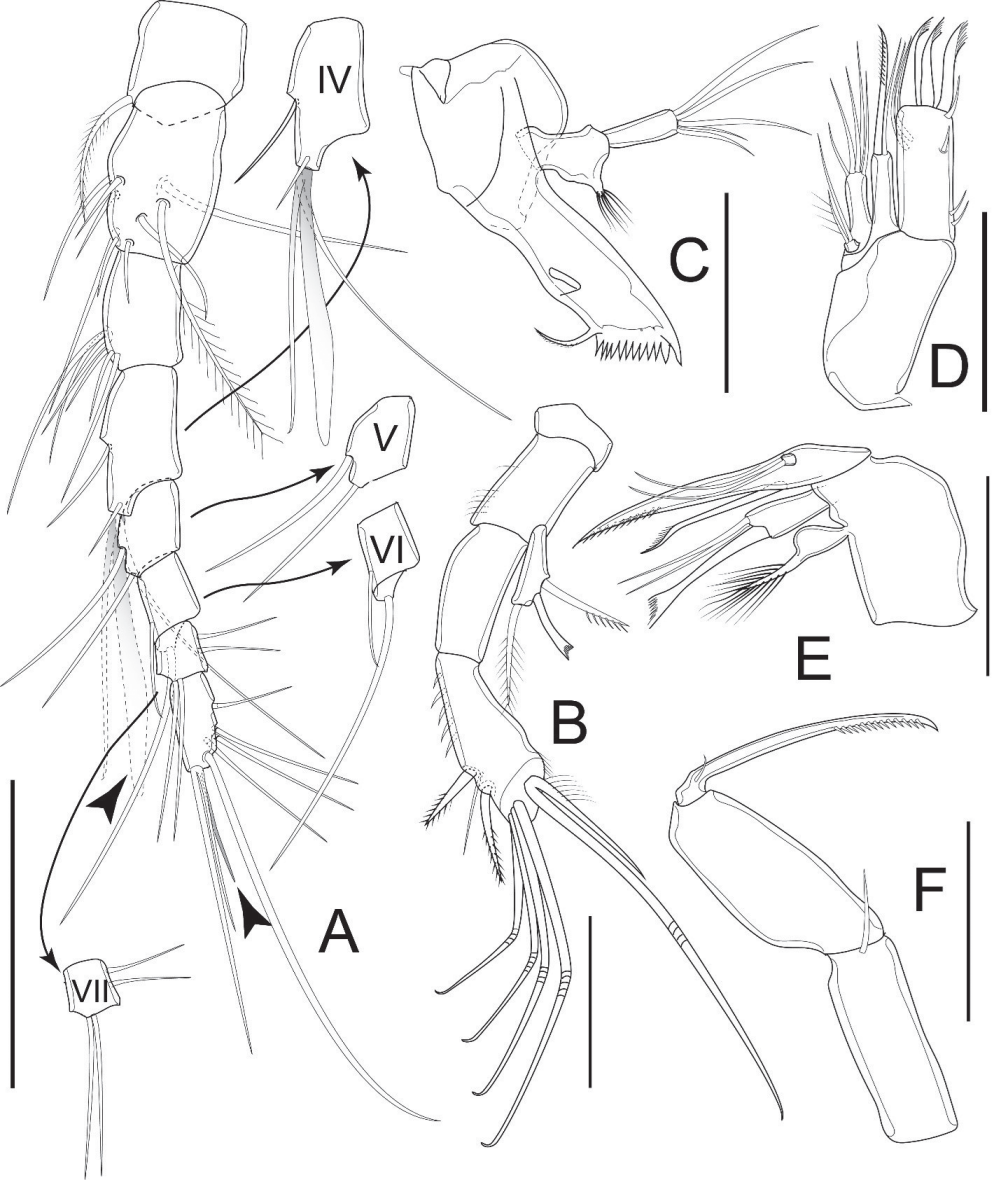
*Nitocrellagrandicaudis* sp. nov., female holotype: **A** antennule **B** antenna **C** mandible **D** maxillule **E** maxilla **F** maxilliped. Arrowheads indicate aesthetascs. Scale bars: 50 μm.

**Figure 4.**
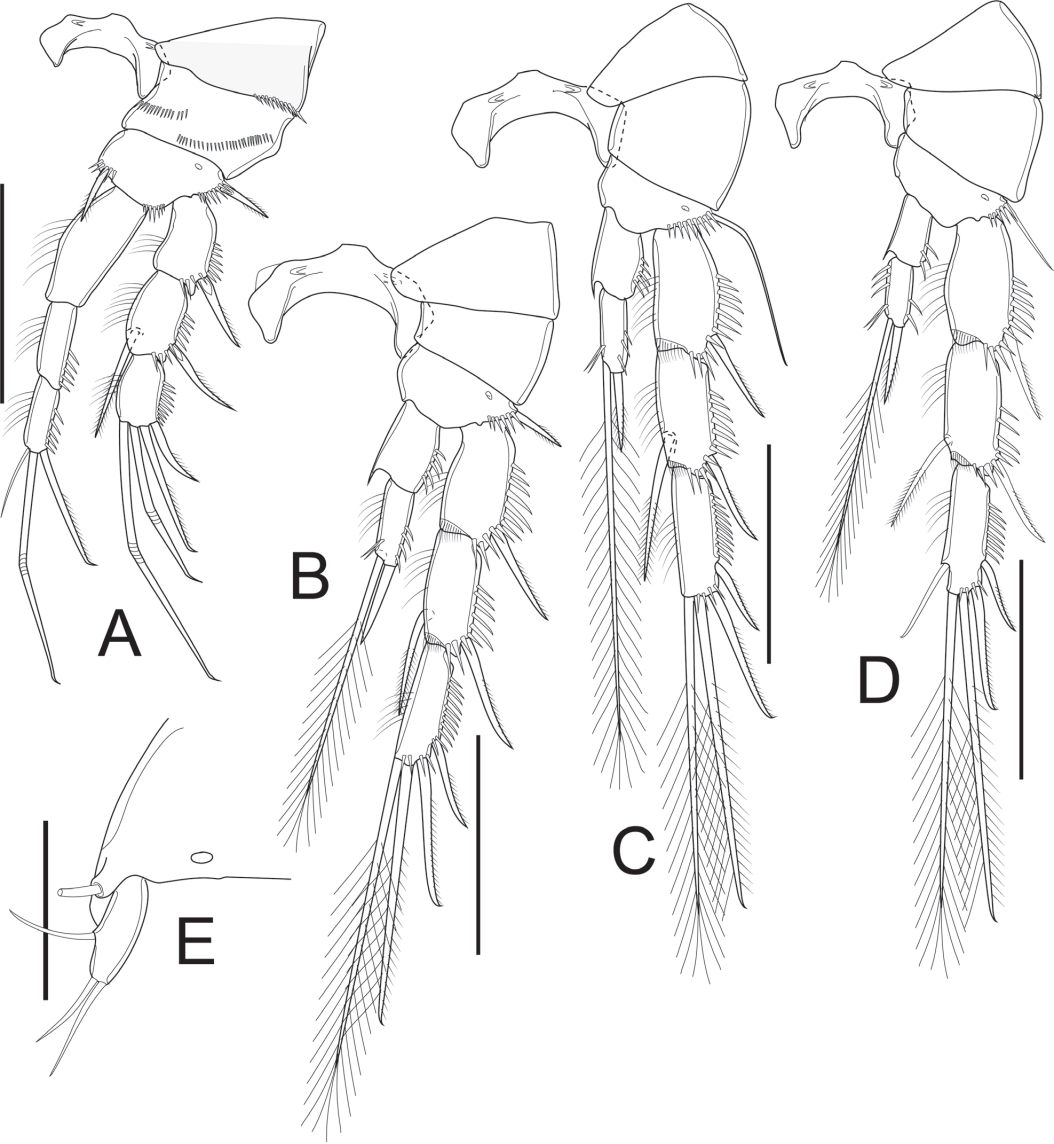
*Nitocrellagrandicaudis* sp. nov., female holotype: **A** P1 **B** P2 **C** P3 **D** P4 **E** P5. Scale bars: 50 μm.

**Figure 5.**
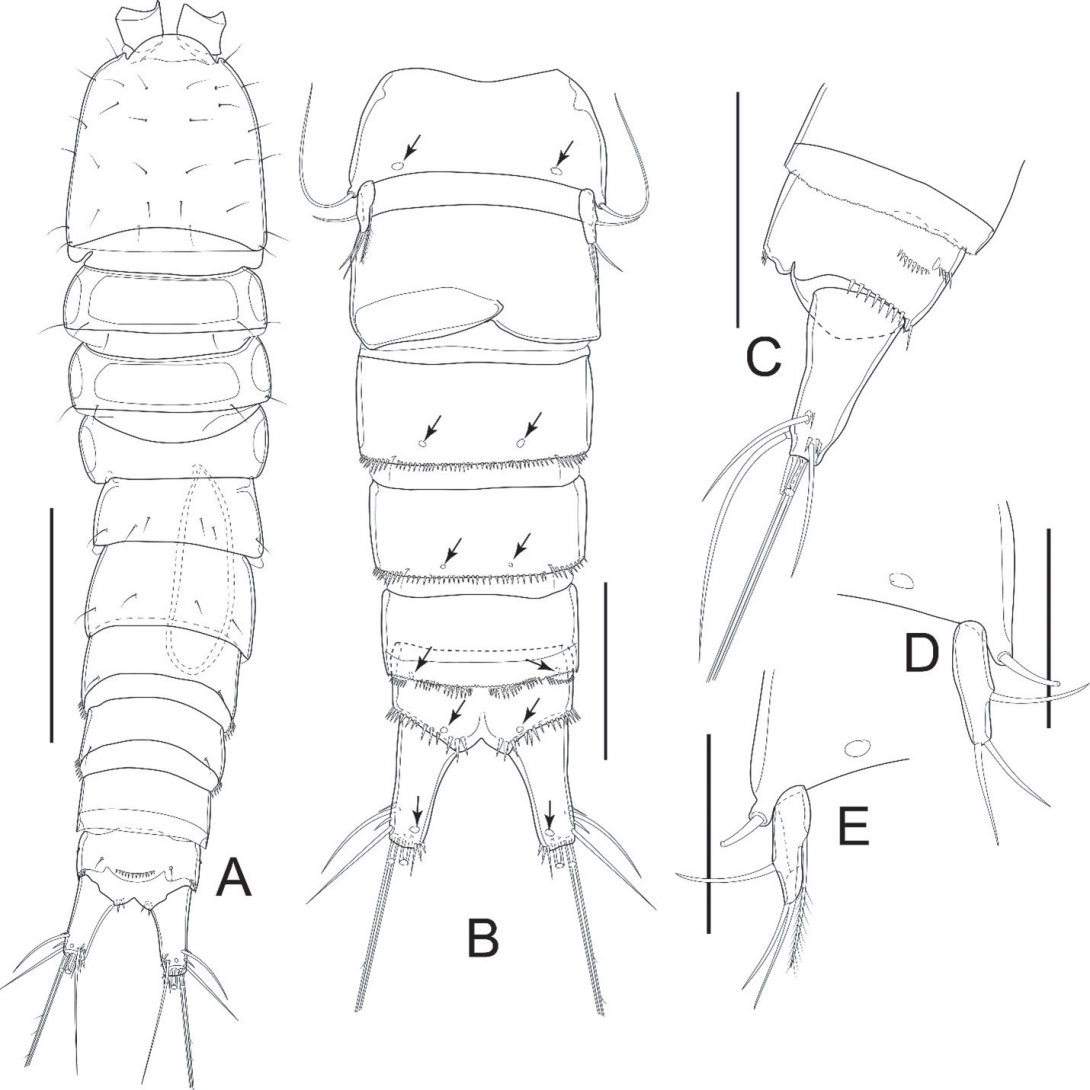
*Nitocrellagrandicaudis* sp. nov., male “allotype”: **A** habitus, dorsal view **B** urosome, ventral view **C** anal somite and caudal rami, lateral view **D** left P5 **E** right P5. Arrows indicate integumental pores. Scale bars: 50 μm (**B–E**).

**Figure 6.**
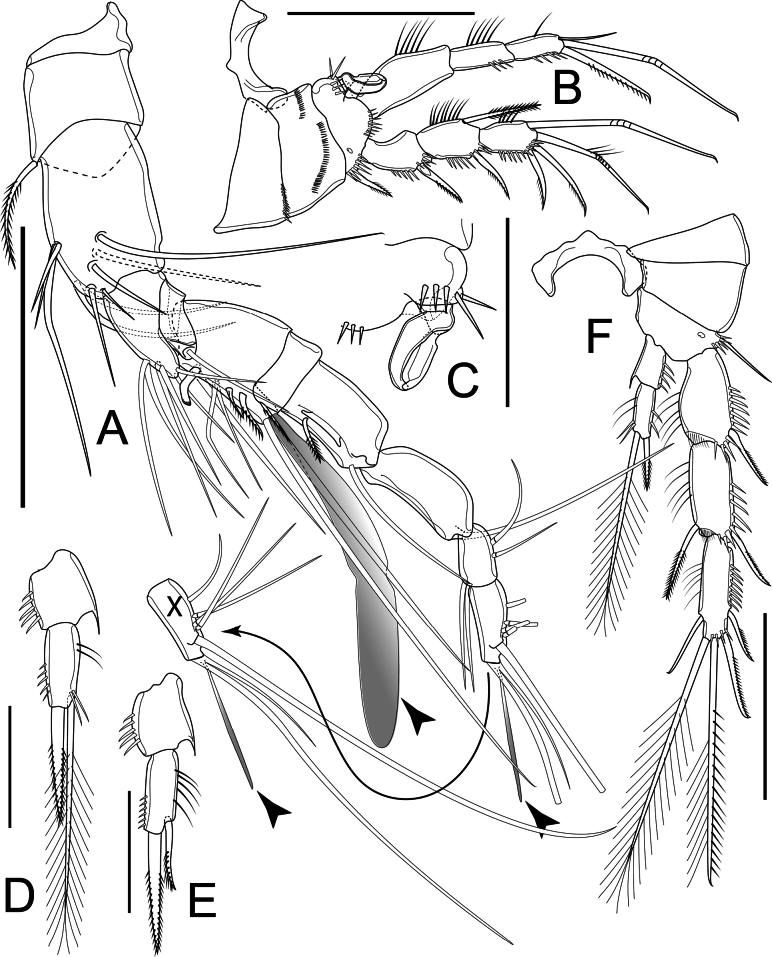
*Nitocrellagrandicaudis* sp. nov., male “allotype”: **A** antennule (arrowheads indicate aesthetascs) **B** P1 **C** P1 basis with complex hook-like transformed seta **D** P2 Enp**E** P3 Enp**F** P4. Arrowheads indicate aesthetascs. Scale bars: 50 μm.

Figs 2 , 3 , 4 (female); Figs 5 , 6 (male)

##### Type material.

***Holotype***: Thailand • 1 ♀ (adult), 470 μm long; northeast Thailand, Loei Province, Nong Hin District, Wat Tham Bodhisattva Temple, 17°05′13.19″N, 101°46′55.59″E, 337 m a.s.l.; 2 April 2023; A. Brancelj, L. Sanoamuang, and N. Sanoamuang leg.; a concrete reservoir at the mouth of a karstic spring; access number: THNHM-IV-20713; completely dissected, mounted on a slide in glycerol, covered with a coverslip and sealed with nail polish. ***Paratype 1* (“*allotype*”)**: Thailand • 1 ♂ (adult), 431 μm long; location, date, and collectors as for holotype; access number: THNHM-IV-20714; completely dissected, mounted on slide in glycerol, covered with coverslip and sealed with nail polish. ***Paratype 2***: Thailand • 1 ♀ (adult); location, date, and collectors as for holotype; access number: THNHM-IV-20715; preserved in 70% ethanol.

##### Additional material.

Thailand • 1 ♂ (adult), 1 ♀ (adult); location, date, and collectors as for holotype; preserved in 70% ethanol; retained in a collection of the first author (CB).

##### Description of adult female.

Total body length (measured from tip of rostrum to posterior margin of caudal rami) ranging from 463 µm to 472 µm (mean = 469 µm; *n* = 3). Sensilla and pores ornamentations as figured (Fig. 2A–C). Habitus subcylindrical, width evenly decreasing from cephalothorax to last urosomite (Fig. 2A, B). Prosome as long as urosome (including caudal rami), comprising cephalothorax and three free pedigerous somites (P2–P4 bearing somites). Naupliar eye not discernible. Integument moderately chitinized, with rectangular integumental sclerite with rounded corners dorsally on first two pedigerous somites; circular integumental windows laterally on all free pedigerous somites. Cephalothorax ~ 1.1× as long as wide and ~ 1/2 as long as prosome, with numerous sensilla dorsally. P2–P4-bearing somites with free margin smooth; each with pair of sensilla near posterior margin dorsally.

Urosome (Fig. 2A–C) comprising P5-bearing somite, genital double-somite, two free urosomites (urosomites 3 and 4) and anal somite with caudal rami; free margins of urosomites 1–3 smooth, urosomite 4 with minute serrulation dorsally. Genital double-somite as long as wide, with ancestral articulation between both somites (Fig. 2A–C). Genital field as shown (Fig. 2C), with single small copulatory pore and one median genital pore covered by P6 fused basal plate; copulatory duct not sclerotized. Urosomites 2 and 3 with continuous row of spinules ventrolaterally on distal margin; a pair of cuticular pores ventrally (Fig. 2B, C). Urosomite 4 lack row of spinules and cuticular pore; with serrated hyaline free margin distally. Anal somite (Fig. 2C–E) with pair of sensilla at base of anal operculum, with robust spinules on distal margin lateroventrally; three groups of spinules on each side ventrally and ventrolaterally; two rows near anterior margin, distal one near caudal ramus. Anal operculum slightly convex, with row of ~ 15 small spinules dorsally along free margin.

Caudal rami (Fig. 2C–F) slightly divergent; semi-elliptical, ~ 1.7× as long as wide, with large dorsal keel and seven setae; with row of few spinules near insertion of seta III, seta VI, and seta VII, respectively; one cuticular pore dorsally and ventrally. Seta I minute, slender, inserted next to seta II; seta II inserted at 3/4 length of ramus, ~ 1/2 length of seta III; seta III inserted on distal outer corner; seta IV and V each with fracture plane, ~ 0.3 and 0.6 of body length, respectively; seta VI short and slender; seta VII articulate, inserted close to ramus tip on inner distal corner.

Antennule (Fig. 3A) eight-segmented; armament formula: I-[1], II-[9], III-[6], IV-[4 + ae], V-[2], VI-[2], VII-[4], VIII-[7 + ae]. Aesthetasc on segment IV reach tip of ultimate segment; that on segment VIII small and slender.

Antenna (Fig. 3B) comprising coxa, basis, one-segmented Exp, two-segmented Enp. Coxa short, unarmed. Basis with row of spinules along inner margin, ~ 2× as long as wide. Exp elongated, with three setae: proximal one spiniform, slender, unilaterally pinnate; inner distal one spiniform, cusped; outer distal one soft, pinnate. Enp-1 ~ 2.5× as long as wide, unarmed; Enp-2 club-shaped, slightly longer than Enp-1, with row of spinules, one slender seta and two pinnate spines along inner margin; six setae apically: one setose, five geniculate; innermost geniculate seta fused to accompanying seta, with spinules only at its base.

Mandible (Fig. 3C) comprising syncoxa, basis, one-segmented Enp. Coxal gnathobase with two robust and eight or nine unicuspid teeth ventrally; one unilaterally pinnate seta dorsally. Basis with group of filaments on tip of expansion at ~ 2/3 length of segment. Enp slim, ~ 3× as long as wide, with five setae apically, decreasing in length.

Maxillule (Fig. 3D) comprising praecoxa with arthrite, coxa, basis and one-segmented Enp. Praecoxa robust, subrectangular, ~ 2× as long as wide; praecoxal arthrite rectangular, ~ 2.5× as long as wide, with seven elements: three cusped spines apically, one spine subapically, one short seta ventrally, two setae equal in length on anterior surface. Coxa with cylindrical endite with one curved spiniform seta and one smooth seta apically. Basis with five setae of which outermost shortest, apically. Enp short and minute, with two setae of which shortest one pilose, apically.

Maxilla (Fig. 3E) comprising syncoxa, allobasis, one-segmented Enp. Syncoxa with two endites: proximal endite with several filaments apically; distal endite with three elements apically of which the proximalmost robust, cusped, two distal ones as smooth and slender bare setae. Allobasis drawn-out into strong claw, with one seta on caudal surface. Enp short, minute, with two smooth setae equal in length apically.

Maxilliped (Fig. 3F) prehensile, three-segmented, comprising syncoxa, basis and Enp. Syncoxa ~ 3× as long as wide, with one short seta on inner distal corner. Basis ~ 2× as long as wide, unarmed. Enp drawn-out into strong claw, unilaterally pinnate distally, with minute seta near base.

P1–P4 comprised of intercoxal sclerite, praecoxa, coxa, basis, and two rami. Armament formula of P1–P4 as in Table 1.

**Table 1.** Armament formula of swimming legs P1–P4 of female and male of *Nitocrellagrandicaudis* sp. nov. (inner-outer seta/spine; inner-apical-outer seta/spine; Arabic numerals - setae, Roman numerals - spines).

Swimming leg	Basis	Exp			Enp		
		1	2	3	1	2	3
P1	I-I	0-I	1-I	0-3-I	0-0	0-0	1-1, I-0
P2	0-I	0-I	1-I	0-2, I-I	0-0	0-1, I-0	
P3	0-1	0-I	1-I	0-2, I-I	0-0	0-1, I-0	
P4	0-1	0-I	1-I	1-2, I-I	0-0	0-1, I-0	

P1 (Fig. 4A) intercoxal sclerite wider than long, concave. Praecoxa triangular, with spinules along outer 1/2 of margin distally. Coxa parallelogram-like, with two rows of spinules on dorsal surface, one near inner margin, additional row along outer margin; unarmed. Basis subtriangular, with cuticular pore on anterior surface; three rows of robust spinules distally of which outermost at base of Exp, median between insertion of rami, inner at base of inner spine; outer and inner spines short, robust, pinnate, similar in length. All segments of Exp with row of spinules along outer margin and outer distal corner. Exp-1 ~ 1.5× as long as wide, outer spine as long as segment bearing it. Exp-2 ~ 1.5× as long as wide; outer spine robust, as long as segment bearing it, inner seta slightly longer than segment bearing it. Exp-3 ~ 1.5× as long as wide, with two geniculate and one unilaterally pinnate seta apically, unilaterally pinnate spine on outer margin as long as segment bearing it. Three-segmented Enp well surpass tip of Exp-3; Enp-1 ~ 2.5× as long as wide, reaching 1/2 length of Exp-2; row of setules along inner margin. Enp-2 ~ 2.5× as long as wide, with row of spinules along outer margin. Enp-3 as long as Enp-2, with row of spinules along outer margin, armed with three elements: innermost slender seta as long as segment bearing it, median geniculate seta long, outermost spiniform seta unilaterally pinnate, ~ 1/2 length of geniculate one.

P2 (Fig. 4B) intercoxal sclerite as in P1, but slightly larger. Praecoxa triangular, bare. Coxa trapezoidal, bare. Basis as in P1 but lack medial spine; row of spinules along outer margin. Three-segmented Exp with spinules ornamentation as in P1. Exp-1 ~ 2× as long as wide. Exp-2 ~ 2.1× as long as wide, inner seta and outer spine as long as segment bearing them. Exp-3 ~ 4× as long as wide, with four elements: two long plumose setae, unequal in length and one unilaterally pinnate spine, as long as segment bearing it apically; lateral unipinnate spiniform seta shorter than segment bearing it on outer margin. Two-segmented Enp reaching proximal third of Exp-2, with spinules along outer margin on both segments; Enp-1 ~ 1.3× as long as wide, bare, with acute extension on distal inner corner. Enp-2 ~ 2× as long as wide, with two elements apically: inner seta long, plumose; outer one smooth spine, as long as segment bearing it.

P3 (Fig. 4C) as in P2, but basis with long bare seta on outer margin.

P4 (Fig. 4D) intercoxal sclerite, praecoxa, coxa, basis, and two proximal Exp segments as in P3, but relatively shorter seta on basis on outer margin. Exp-3 with five elements: spine on outer margin relatively thin, shorter than segment bearing it; two plumose setae unequal in length and unilaterally pinnate seta apically; seta on inner margin smooth. Enp as in P2 and P3, but relatively slender, reaching distal margin of Exp-2 at most; Enp-1 ~ 2× as long as wide; Enp-2 as in P2 and P3 but with robust spinules on inner and outer margin.

P5 (Figs 2C, 4E) with baseoendopod completely incorporated into somite with long, smooth slender seta on outer margin; one-segmented Exp. Exp elliptical, ~ 3× as long as wide, with one smooth seta on outer margin; two smooth setae apically, inner seta slightly longer than outer one.

P6 (Fig. 2C) reduced, represented by fused basal plate; on both sides with one minute seta and knob-like extension on distolateral margin.

##### Description of adult male.

Total body length, excluding caudal setae, 428 and 431 µm (mean = 430 µm; *n* = 2). Sensilla and pore ornamentations as figured (Fig. 5A, B). Prosome as long as urosome (Fig. 5A). Naupliar eye and integument as in female. Cephalothorax ~ 1.1× as long as wide and ~ 1/2 length of prosome, with numerous sensilla. Other characteristics of prosoma somites as in female.

Urosome (Fig. 5A, B) comprising P5-bearing somite, genital somite, three free urosomites and anal somite with caudal rami; free margins of all somites smooth dorsally. P5-bearing somite as in female. Genital somite with three pairs of subdistal sensilla dorsally (Fig. 5A). Three subsequent urosomites, anal somite and anal operculum as in female.

Caudal rami (Fig. 5B, C) sexually dimorphic, slightly divergent, subconical, ~ 2.5× as long as wide; ornamentation as in female but without dorsal keel.

Antennule (Fig. 6A) ten-segmented, geniculate, with geniculation between segments VII and VIII. Armament formula: I-[1], II-[10], III-[6], IV-[1], V-[8 + ae], VI-[1], VII-[2], VIII-[1], IX-[4], X-[8 + ae]. Segment V with proximalmost inner seta modified into strong curved spiniform element. Aesthetasc on segment V long, reaching beyond tip of segment X of antennule.

Antenna, mandible, maxillule, maxilla, and maxilliped as in female.

P1 (Fig. 6B) as in female, but basis with complex hook-like transformed seta on inner margin (Fig. 6C).

P2 as in female, except with relatively larger outer spine apically on Enp-2 (Fig. 6D).

P3 as in female, but Enp-2 with robust outer pinnate spine, longer than segment bearing it and shorter inner seta with feather-like tip apically (Fig. 6E).

P4 (Fig. 6F) and P5 (Fig. 5D, E) as in female.

P6 (Fig. 5B). Left and right legs form simple plate, unarmed, with smooth distal margin; asymmetrical; right leg reduced into simple plate, operculiform; left leg completely incorporated into somite.

##### Variability.

The “allotype” right P5 Exp with an additional plumose seta on the inner margin (Fig. 5E) is present, but absent in left P5 (Fig. 5D).

##### Etymology.

The specific name is an allusion to a ridge on the dorsal side of a female caudal ramus, producing a high lateral profile. The name is an adjective in the nominative singular, feminine gender.

#### ﻿ Nitocrella thailandensis sp. nov.

Taxon classificationAnimaliaHarpacticoidaAmeiridae

D4523366-0C14-5D7E-88FE-68A1049CDD33

https://zoobank.org/D054B982-42AE-499A-99BD-60DE73504B46

**Figure 7.**
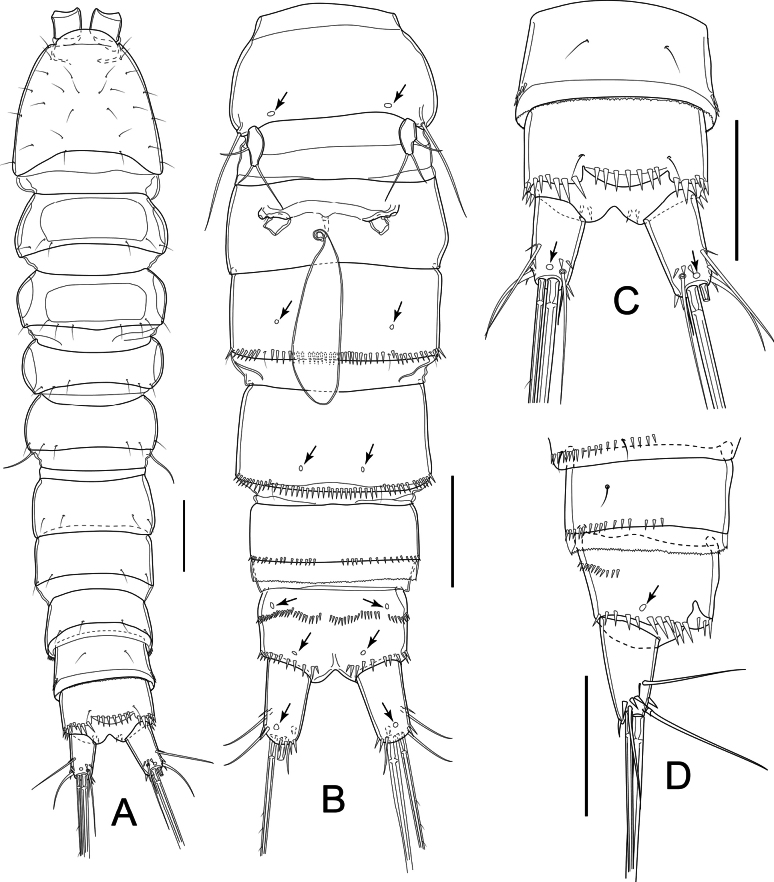
*Nitocrellathailandensis* sp. nov., female holotype: **A** habitus, dorsal view **B** urosome, ventral view **C** urosomite 4, anal somite and caudal rami, dorsal view **D** urosomite 4, anal somite and caudal rami, lateral view. Arrows indicate integumental pores. Scale bars: 50 μm.

**Figure 8.**
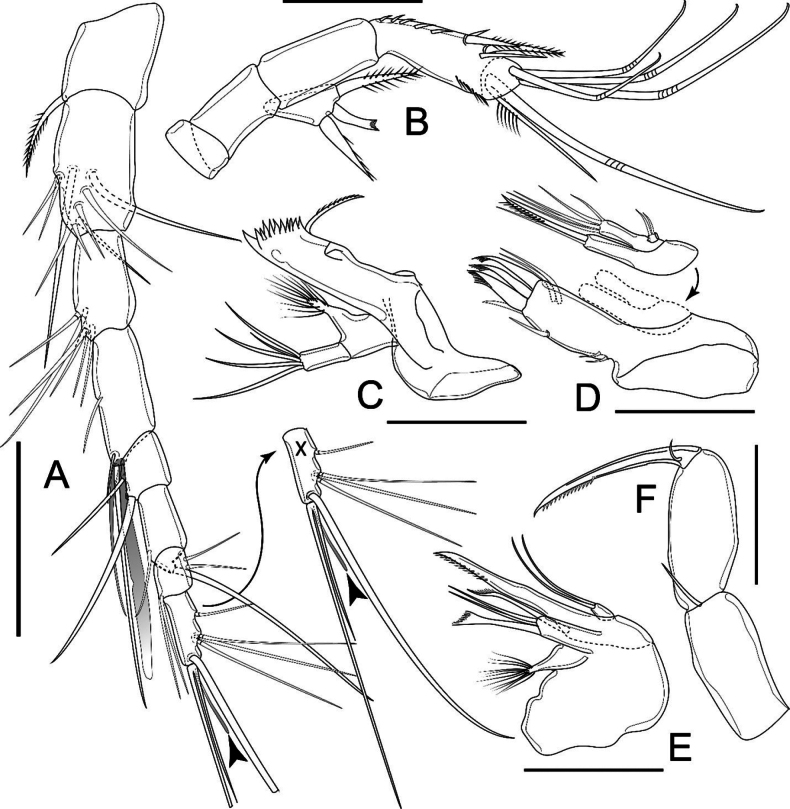
*Nitocrellathailandensis* sp. nov., female holotype: **A** antennule **B** antenna **C** mandible **D** maxillule **E** maxilla **F** maxilliped. Arrowheads indicate aesthetascs. Scale bars: 50 μm.

**Figure 9.**
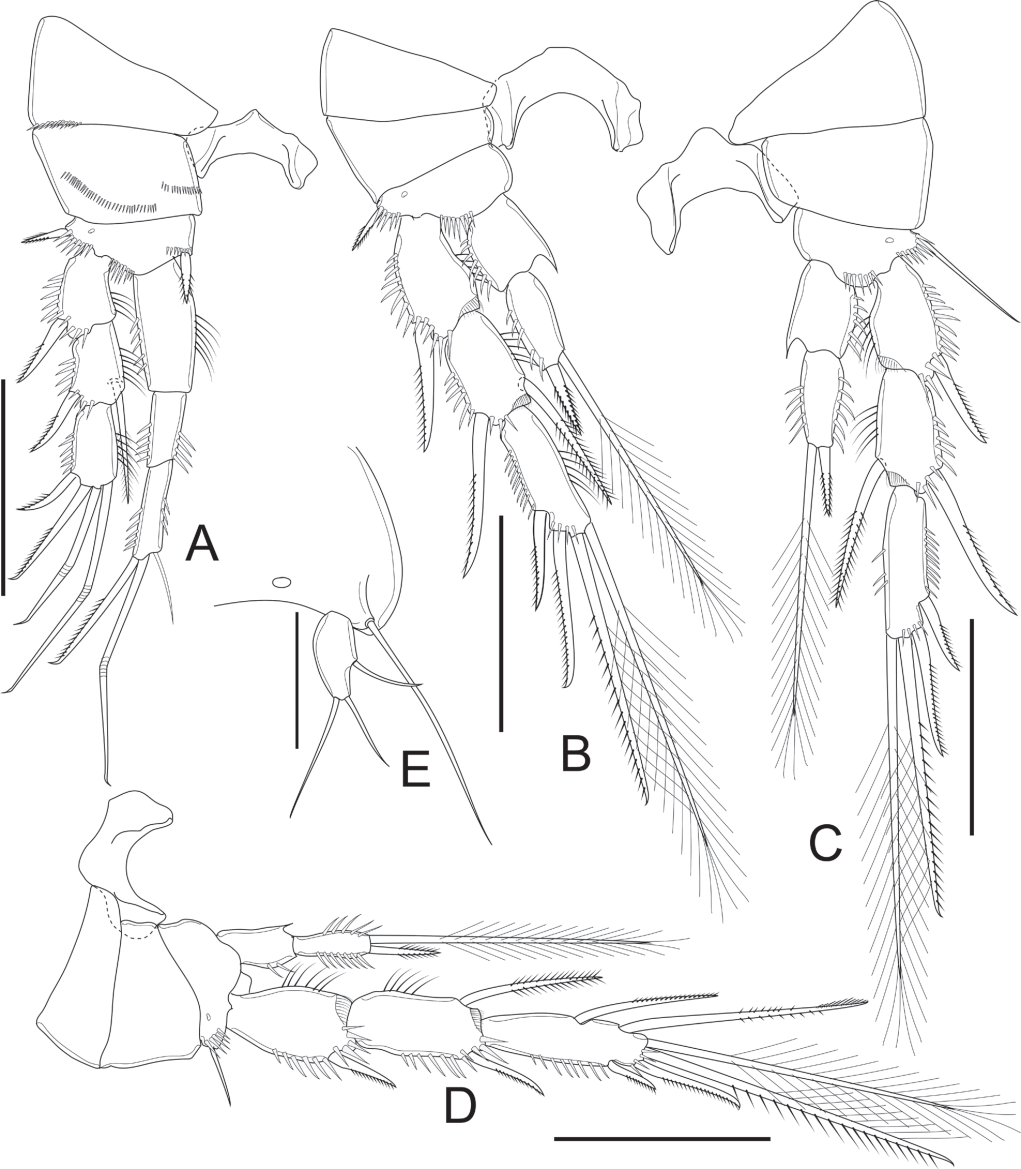
*Nitocrellathailandensis* sp. nov., female holotype: **A** P1 **B** P2 **C** P3 **D** P4 **E** P5. Scale bars: 50 μm.

**Figure 10.**
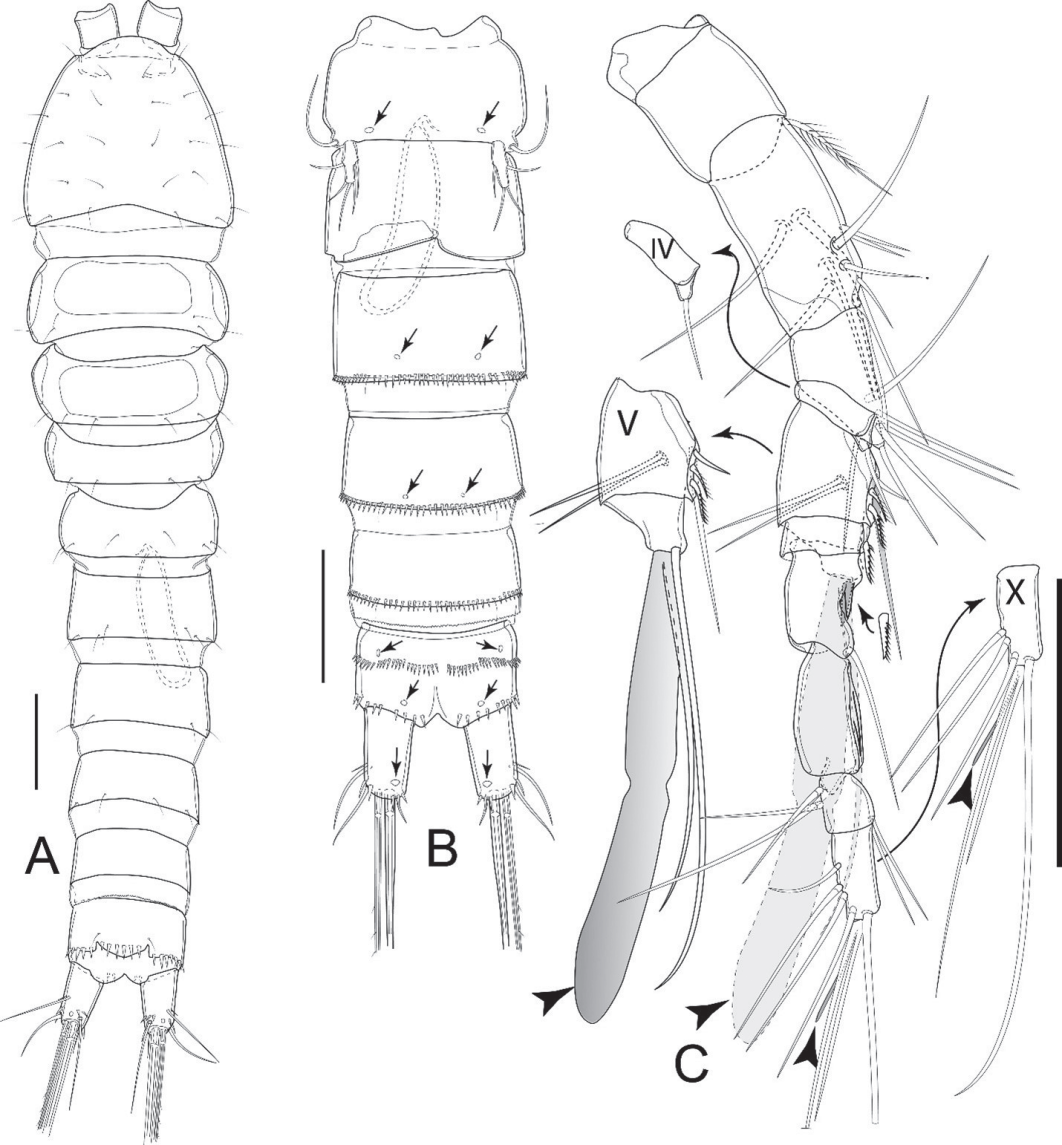
*Nitocrellathailandensis* sp. nov., male “allotype”: **A** habitus, dorsal view **B** urosome, ventral view **C** antennule. Arrows and arrowheads indicate integumental pores and aesthetascs, respectively. Scale bars: 50 μm.

**Figure 11.**
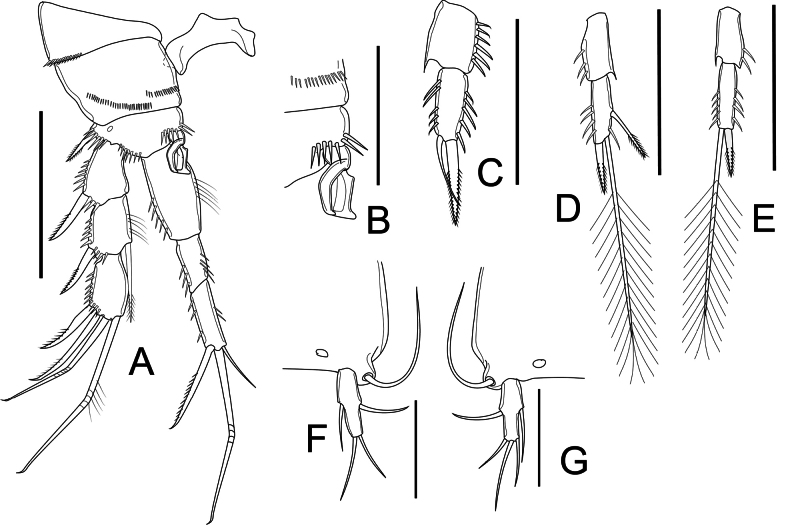
*Nitocrellathailandensis* sp. nov., male “allotype”: **A** right P1 **B** P1 basis with complex hook-like transformed seta **C** P3 Enp**D** right P4 Enp**E** left P4 Enp**F** left P5 **G** right P5. Scale bars: 50 μm.

Figs 7 , 8 , 9 (female); Figs 10 , 11 (male)

##### Type material.

***Holotype***: Thailand • 1 ♀ (adult), 595 μm long; northeast Thailand, Loei Province, Nong Hin District, Wat Tham Bodhisattva Temple, 17°05′13.19″N, 101°46′55.59″E, 337 m a.s.l.; 2 April 2023, A. Brancelj, L. Sanoamuang and N. Sanoamuang leg.; a concrete reservoir at mouth of karstic spring; access number: THNHM-IV-20716; completely dissected, mounted on a slide in glycerol, covered with coverslip and sealed with nail polish. ***Paratype 1* (“*allotype*”)**: Thailand • 1 ♂ (adult), 564 μm long; location, date, and collectors as for holotype; access number: THNHM-IV-20717; completely dissected, mounted on slide in glycerol, covered with coverslip and sealed with nail polish. ***Paratype 2***: Thailand • 1 ♀ (adult); location, date, and collectors as for holotype; access number: THNHM-IV-20718; preserved in 70% ethanol.

##### Additional material.

Thailand • 1 ♂ (adult), 1 ♀ (adult); location, date, and collectors as for holotype; preserved in 70% ethanol; retained in a collection of the first author (CB).

##### Description of adult female.

Total body length (measured from tip of rostrum to posterior margin of caudal rami) ranging from 539 µm to 595 µm (mean = 577 µm; *n* = 3). Sensilla and pore ornamentations as figured (Fig. 7A–D). Habitus subcylindrical, width evenly decreasing from cephalothorax to last urosomite (Fig. 7A). Prosome slightly shorter than urosome (including caudal rami), comprising cephalothorax and three free pedigerous somites (P2–P4-bearing somites). Naupliar eye not discernible. Integument moderately chitinized, with rectangular integumental sclerite with rounded corners dorsally on first two pedigerous somites; circular integumental windows laterally on all free pedigerous somites. Cephalothorax ~ 0.9 as long as wide and ~ 0.8 as long as prosome, with numerous sensilla. All free prosomal somites with subdistal sensilla near posterior margin.

Urosome (Fig. 7A, B) comprising P5-bearing somite, genital double-somite, two free urosomites and anal somite with caudal rami; free margins of urosomites 1–3 smooth, urosomite 4 with minute serrulation. Genital double-somite as long as wide, with ancestral articulation between both somites. Genital field as shown (Fig. 7B), with single small copulatory pore; one medial genital pore partly covered by P6 fused basal plate; copulatory duct not sclerotized. Urosomites 2–4 with continuous row of spinules lateroventrally on distal margin but interrupted medially on urosomite 4; pair of cuticular pores ventrally on free urosomites 2 and 3.

Anal somite (Fig. 7B–D) with pair of sensilla at base of anal operculum; three or four robust spinules dorsolaterally on distal free margin continuing in row of moderate spinules laterally and ventrally; row of spinules at 1/2 length of somite, organized in three groups ventrally: medial and two lateral; pair of cuticular pores on anterior and posterior region of segment ventrally. Anal operculum slightly convex, not reaching tip of anal segment, with six or seven robust spinules along free margin (Fig. 7C).

Caudal rami (Fig. 7B–D) slightly divergent; subconical, ~ 1.7× as long as wide, with cuticular pore dorsally and ventrally; with seven setae; three robust spinules dorsally, near insertion of seta VII, three laterally near insertion of seta III, two near insertion of seta VI. Seta I minute, slender, inserted just below seta II; seta II inserted at 2/3 length of ramus, ~ 1.5× length of seta III; seta III inserted on distal outer corner, ~ 0.8 length of ramus; seta IV and V each with fracture plane, ~ 0.3 and 0.6 of body length, respectively; seta VI short and slender; seta VII articulate, inserted close to ramus tip on inner distal corner, ~ 1.3× as long as ramus.

Antennule (Fig. 8A) eight-segmented; armament formula: I-[1], II-[9], III-[6], IV-[4 + ae], V-[2], VI-[2], VII-[4], VIII-[7 + ae]. Aesthetasc on segment IV reaching tip of ultimate segment; ae on segment VIII small and slender, as long as segment bearing it.

Antenna (Fig. 8B) comprising coxa, basis, one-segmented Exp, two-segmented Enp. Coxa short, unarmed. Basis unarmed, ~ 2× as long as wide. Exp triangular, with three robust setae: proximal one spiniform, unilaterally pinnate; inner distal one spiniform, cuspid; distal one stout, pinnate. Enp-1 ~ 2.5× as long as wide, unarmed; Enp-2 slightly longer than Enp-1, with row of spinules plus one slender seta and two pinnate spines along inner margin, all short and robust; six setae apically: one setose and five geniculate setae, unequal in length; innermost geniculate seta fused to accompanying setose seta with spinules only at its base.

Mandible (Fig. 8C) comprising coxa, basis, one-segmented Enp. Coxal gnathobase with one robust and eight or nine unicuspid teeth ventrally; one unilaterally pinnate seta dorsally. Basis with group of filaments on tip of expansion at ~ 2/3 length of segment. Enp slim, ~ 2.5× as long as wide, with five apical setae, four equal in length, one shorter.

Maxillule (Fig. 8D) comprising praecoxa with arthrite, coxa, basis and one-segmented Enp. Praecoxa robust, subrectangular, ~ 2× as long as wide; praecoxal arthrite rectangular, ~ 2.5× as long as wide, with seven elements: three robust cuspid spines apically, one subapically, one short seta ventrally; two setae equal in length on anterior surface. Coxa with cylindrical endite with one curved, unilaterally pinnate spiniform seta and one smooth seta apically. Basis with five setae apically of which outermost shortest. Enp short and minute, with two setae apically, unequal in length.

Maxilla (Fig. 8E) comprising syncoxa, allobasis, one-segmented Enp. Syncoxa with two endites: proximal one with a patch of filaments apically; distal one with three elements apically of which the proximalmost robust, cusped; two distal ones as smooth and slender bare setae. Allobasis drawn-out into strong claw, with one seta on caudal surface. Enp minute, ~ 1.5× as long as wide, with two smooth setae equal in length apically.

Maxilliped (Fig. 8F) prehensile, three-segmented, comprising syncoxa, basis, and Enp. Syncoxa ~ 2.1× as long as wide, with one short seta on inner distal corner. Basis ~ 2× as long as wide, unarmed. Enp drawn-out into long robust claw, with spinules distally, minute seta near its base.

P1–P4 comprised of intercoxal sclerite, praecoxa, coxa, basis, and two rami. Armament formula of P1–P4 as in Table 2.

**Table 2.** Armament formula of swimming legs P1–P4 of female and male of *Nitocrellathailandensis* sp. nov. (inner-outer seta/spine; inner-apical-outer seta/spine; Arabic numerals - setae, Roman numerals - spines).

Swimming leg	Basis	Exp			Enp		
		1	2	3	1	2	3
P1	I-I	0-I	1-I	0-3-I	0-0	0-0	1-1, I-0
P2	0-I	0-I	1-I	0-2, I-I	0-0	0-1, I-0	
P3	0-1	0-I	1-I	0-2, I-I	0-0	0-1, I-0	
P4	0-1	0-I	1-I	2-2, I-I	0-0	0-1, I-0	

P1 (Fig. 9A) intercoxal sclerite wider than long, concave. Praecoxa triangular, with spinules along outer distal margin. Coxa parallelogram-like, with two rows of spinules on dorsal surface, one near inner margin, additional row along outer margin. Basis subtriangular, with cuticular pore on anterior surface, three rows of robust spinules of which inner at base of inner spine, median between insertion of rami, outer along the outer margin; inner and outer spines pinnate, inner spine reaching proximal third of Enp-1. All Exp segments with row of spinules along outer margin and on outer distal corner; Exp-1 ~ 1.8× as long as wide, with outer spine as long as segment bearing it. Exp-2 ~ 1.8× as long as wide with inner seta and outer spine as long as segment bearing them. Exp-3 ~ 1.8× as long as wide with one unipinnate seta, two geniculate setae unequal in length apically; outer spine as long as segment bearing it. Enp well surpassing tip of Exp. Enp-1 ~ 2.5× as long as wide, well overreaching mid of Exp-2. Enp-2 ~ 2.5× as long as wide, with row of spinules along outer margin. Enp-3 ~ 3× as long as wide, ~ 1.5× as long as Enp-2, with row of spinules along outer margin, with three elements: a slender seta subapically on inner margin, and a geniculate seta and a spine apically.

P2 (Fig. 9B) intercoxal sclerite as in P1 but larger. Praecoxa triangular, bare. Coxa trapezoidal, bare. Basis as in P1 but lack medial spine, with two rows of spinules, one along outer margin, one between insertion of Exp and Enp. Exp-1 ~ 1.5× as long as wide, outer spine as long as segment bearing it, robust. Exp-2 ~ 1.5× as long as wide, with inner seta and outer spine ~ 1.7× longer than segment bearing them. Exp-3 ~ 3× as long as wide; three elements apically: one plumose seta, one plumose on one side and pinnate on other side, outer unipinnate seta as long as segment bearing it. Enp reaching 3/4 length of Exp-2. Enp-1 as long as wide, bare, with acute extension on distal inner corner. Endp-2 ~ 2.5× as long as wide; inner apical plumose seta longer than Enp; outer apical spine robust, shorter than segment bearing it.

P3 (Fig. 9C) as in P2 but basis with long bare seta on outer margin; Enp-2 with robust spinules on both margins.

P4 (Fig. 9D) intercoxal sclerite, praecoxa, coxa, basis, and two proximal Exp segments as in P3, with relatively shorter lateral seta on basis. Exp-3 ~ 3.5× as long as wide; along inner margin two spiniform setae unequal in length, longer one ~ 1.5× length of segment bearing it; one plumose seta, other one plumose on one side and pinnate on other side, equal in length apically; outer spine apically shorter than segment bearing it; outer lateral spine short. Enp as in P3, but relatively slender, just reaching proximal margin of Exp-2.

P5 (Fig. 9E) with baseoendopod completely incorporated into somite, with long bare slender seta on outer margin; one segmented Exp. Exp elliptical, ~ 2× as long as wide; inner seta ~ 2× as long as outer one; two smooth setae apically; one smooth seta on outer margin.

P6 (Fig. 7B) reduced, represented by fused basal plate; on both sides one minute seta and knob-like extension on distolateral margin.

##### Description of adult male.

Total body length, excluding caudal setae, 564 and 562 µm (*n* = 2). Sensilla and pore ornamentation as figured (Fig. 10A, B). Prosome as long as urosome (Fig. 10A). Naupliar eye and integument ornamentation as in female. Cephalothorax ~ 0.9× as long as wide, ~ 1/2 length of prosome, with numerous sensilla. Other characteristics of prosoma somites as in female, but presence of continuous row of spinules on urosomite 5 ventrally (Fig. 10B).

Urosome (Fig. 10A, B) comprising P5-bearing somite, genital somite, three free urosomites and anal somite with caudal rami; free margins of urosomites 1–4 smooth, urosomite 5 with minute serrulation. P5-bearing somite as in female. Genital somite with three pairs of subdistal sensilla dorsally, ~ 1/2 as long as wide (Fig. 10A, B). Three subsequent urosomites, anal somite, caudal rami, and anal operculum as in female.

Antennule (Fig. 10C) ten-segmented, geniculate; geniculation between segments VII and VIII. Armament formula: I-[1], II-[10], III-[6], IV-[1], V-[8 + ae], VI-[1], VII-[2], VIII-[1], IX-[4], X-[7 + ae]. Aesthetasc on segment V large, well over-rich tip of antennule; that of ultimate segment small and slender.

Antenna and mouthparts as in female.

P1 (Fig. 11A, B) as in female, but basis with complex hook-like transformed seta on inner margin.

P2, P3, and P4 as in female, but P3 Enp-2 (Fig. 11C) with large outer spine and short inner seta apically.

P5 (Fig. 11G) with baseoendopod completely incorporated into somite with long, bare slender seta on outer margin; one segmented Exp. Exp elongated, ~ 3× as long as wide, with one smooth seta on inner margin, two smooth setae apically, outer seta as long as outer one.

P6 (Fig. 10B) left and right leg form simple plate, asymmetrical, bare, with smooth distal margins. Right leg reduced into simple plate, operculiform; left leg completely incorporated into somite.

##### Variability.

The “allotype” bears an additional pinnate seta on the inner margin on the right P4 Enp-2 (Fig. 11D) and an additional inner seta on the right P5 Exp apically (Fig. 11G) compared with relevant left-side swimming legs.

##### Etymology.

The specific name is formed from Thailand, alluding to the name of the country where the new species was discovered. The name is an adjective in the nominative singular, gender feminine.

### ﻿Differential diagnosis of the two new species with remarks on closest congeners

Based on the taxonomic definition of the genus sensu Petkovski (1976), both new species belong to the genus *Nitocrella*, according to the following characters: (1) P1 with three-segmented Enp; (2) P1 Exp-2 with inner seta; (3) P2–P4 with two-segmented Enp; (4) P2–P4 lacking an inner element on Exp-1; (5) P2 with two outer elements on Exp-3. The hook-like shape of the modified seta on the basis of the male P1 is characteristic for the family Ameiridae (Galassi and De Laurentiis 1997; Karanovic 2000; Tang and Eberhard 2016) but was not mentioned by Petkovski (1976) when he established the new definition of the genus. At the same time, similarity of the complex transformed hook-like seta on the basis of males P1 indicates close phylogenetic relationships between both species within the genus.

The absence of a proximal inner seta on P4 Exp-3 designated *N.grandicaudis* sp. nov. as a member of the *chappuisi* group, while *N.thailandensis* sp. nov., with six elements on P4 Exp-3, belongs to the *vasconica* group (Petkovski 1976). Although both new species were collected from the same locality, they are easily distinguished from each other based on several characteristics: (1) the shape of the female caudal ramus, which is semi-elliptical, ~ 1.7× as long as wide, with a large dorsal keel in *N.grandicaudis* sp. nov., but subconical, ~ 1.7× as long as wide, with cuticular pores dorsally and ventrally in *N.thailandensis* sp. nov.; (2) the armament of P4 Exp-3 with five elements in *N.grandicaudis* sp. nov., but six elements in *N.thailandensis* sp. nov.; (3) the free margin of the anal operculum in *N.grandicaudis* sp. nov. with ~ 15 small spinules, but six or seven robust spinules in *N.thailandensis* sp. nov.; (4) male P5 with three or four elements in *N.grandicaudis* sp. nov., but four or five elements in *N.thailandensis* sp. nov. In addition, *Nitocrellathailandensis* sp. nov. differs from *N.grandicaudis* sp. nov. in the shape of the transformed hook-like seta on a male P1 basis with an expansion at the tip of the hook-like transformed seta, which is lacking in the former one. More details are provided in Table 3.

**Table 3.** Morphological comparison between two new species, *Nitocrellagrandicaudis* sp. nov. and *N.thailandensis* sp. nov., from Thailand and their most similar congeners. NA = no data available, * = *N.chappuisi* group; *^#^* = *N.vasconica* group.

Character	*Nitocrellagrandicaudis sp. nov.	*Nitocrellajaponica Miura, 1962	^#^*Nitocrellathailandensis* sp. nov.	^#^*Nitocrellayokotai* Miura, 1962	*^#^Nitocrellamotasai* Petkovski, 1976
**Female**					
Ornamentation of basis of mandible	with group of spinules	NA	with group of spinules	NA	NA
Armament of basis of mandible	absent	absent	absent	absent	NA
Proximal endite of maxilla	present	absent	present	absent	NA
Length/width ratio of caudal ramus	~ 1.7	~ 3	~ 1.7	~ 3	~ 1
Ornamentation on free margin of anal operculum	~ 15 minutes spinules	minute spinules	6 or 7 robust spinules	minute spinules	4 robust spinules
Number of setae on antennary Exp	3	1	3	3	3
Seta/spine formula of P1–P4 Exp-2	1.1.1.1	1.1.1.1	1.1.1.1	1.1.1.1	1.1.1.1
Seta/spine formula of P2–P4 Exp-3	4.4.5	4.4.5	4.4.6	4.4.6	4.4.6
Inner seta on P1 Enp-2	absent	absent	absent	absent	absent
Seta/spine formula of P1–P4 Enp-1	0.0.0.0	0.0.0.0	0.0.0.0	0.0.0.0	0.0.0.0
Seta/spine formula of P2–P4 Enp-2	2.2.2	2.2.2	2.2.2	2.2.2	2.2.2
Number of setae onP5 Exp	3	3	3	3	4
**Male**					
Relative length of caudal rami	~ 2×	~ 3×	~ 1.8×	~ 3×	~ 1×
Shape of inner element on P1 basis	complex hook-like transformed seta	NA	complex hook-like transformed seta	NA	NA
Number of elements on P5 Exp	0/3	0/5	0/4	0/4	0/5
Habitat	karst spring	well	karst spring	well	hyporheic zone
Distribution	Thailand	Japan	Thailand	Japan	Cuba

Compared with other species of the *chappuisi* group, *N.grandicaudis* sp. nov. most resembles *N.japonica* Miura, 1962. They share characteristics of the armament formula of P1–P4 Exp-3 as 4.4.4.5, the armament formula of P2–P4 Enp-2 as 2.2.2, the P1–P4 Enp-1 without armament elements, and the P5 baseoendopodal lobe completely incorporated to the somite bearing it. However, *N.grandicaudis* sp. nov. differs from the Japanese congener in the relative length of the caudal ramus and the number of setae on the male P5 Exp: three in *N.grandicaudis* sp. nov. and five in *N.japonica*. It also differs from *N.japonica* by an evident crest on the female caudal ramus dorsally. The species also exhibits sexual dimorphism on the caudal rami, which is not common in the family Ameiridae.

*Nitocrellathailandensis* sp. nov. belongs to the *vasconica* group, as it bears six elements on P4 Exp-3 (Petkovski 1976). Among representatives of the *vasconica* group, *N.thailandensis* sp. nov. most resembles *N.motasai* Petkovski, 1976, in the characteristics of (1) the completely reduced P5 baseoendopodal lobe in both sexes; (2) the same number of armament elements on P2–P4 Exp-3 and Enp-2; (3) the absence of the inner seta on P1–P4 Enp-1; and (4) the ornamentation of the anal operculum (Table 3). However, the number of armament elements of P5 Exp is different. The new species bears a P5 Exp with three setae in the female and four setae in the male, but there are four and five setae, respectively, in *N.motasai*. The new species also shows a close affinity to *N.yokotai* Miura, 1962, having three setae on the P5 Exp in both sexes, but the former has relatively shorter caudal rami and more robust spinules on the anal operculum.

## ﻿Discussion

Finding two new species in a saturated karstic aquifer suggests a rich assemblage of not-yet-discovered species of subterranean copepods in Thailand (see also Boonyanusith et al. 2024). Of 34 copepod species described so far from the caves and other subterranean habitats in Thailand, *N.grandicaudis* sp. nov. and *N.thailandensis* sp. nov. are the third and fourth species of the Copepoda that have been discovered from the springs, following *Fiercyclopssolaris* Boonyanusith, Brancelj & Sanoamuang, 2013 (Cyclopoida, Cyclopidae Dana, 1846) and *Elaphoidellapropecabezasi* Boonyanusith, Brancelj & Sanoamuang, 2024 (Harpacticoida, Canthocamptidae Sars, 1906), from the western and the northeastern regions, respectively (Boonyanusith et al. 2013, 2024). These two new species are the first records of the genus *Nitocrella* in Thailand.

The two new species from Thailand differ from their congeners based on literature descriptions (figures or text) in three additional microcharacters:

the inner element of the male P1 basis is transformed into the uni-appendicular structure, which is either unbranched in *N.ensifera* Cottarelli, Bruno & Berera, 2007; *N.galassiae* Ranga Reddy & Totakura, 2016; *N.karanovici* Tang & Eberhard, 2016; *N.kunzi* Galassi & De Laurentiis, 1997; while both new species have rather complex hook-like transformed seta;

the basis of the mandible is unarmed in *N.hypogaea* Shen & Tai, 1973; *N.tirolensis* Kiefer, 1963; *N.stetinai* Štêrba, 1973; *N.neutra* Kiefer, 1933; *N.ensifera*; *N.knotti*; *N.karanovici*; *N.kunzi*; *N.delayi*; *N.kirgizica*; *N.kyzylkumica*; and *N.monchenkoi*; armed with one subapical seta, as in *N.pescei*, *N.galassiae*, and *N.longa*, but with a group of filaments at the tip in both new species.

the maxilla with one endite on syncoxa in *N.beatricis* Cottarelli & Bruno, 1993; *N.galassiae*; *N.knotti*; *N.delayi*; or with two endites, of which the proximal one bears two apical elements as in *N.pescei*, *N.kunzi*, and *N.longa*, but in both two new species there are two endites, of which the proximal one has a modified apical spine not well defined at the base and a patch of filaments apically; the distal endite has three elements apically. However, a not well-defined base of apical spine and a patch of filaments apically of endite were reported also for *Nitocrellopsisrouchi* Galassi, De Laurentiis & Dole-Olivier, 1999 (Galassi et al. 1999).

Regarding the ornamentation of the basis of the mandible and the proximal endite of the maxilla, a characteristic of the new species, although very similar in structure, has been found previously in *N.rouchi*. In that species, Galassi et al. (1999) provided the description of the armament and ornamentation of the maxilla proximal endite as “*with one modified seta not defined at base, densely filamentous in distal third*,” and the illustration clearly shows the long, wavy, filamentous structures positioned at the tip of the proximal endite. This matches well with the characteristics of the filaments described for both new *Nitocrella* species. Although the characteristics have not been compared to all congeners because of an incomplete description or illustration of mouthparts and the inner transformed element on the male P1 basis in many species, it is reasonable to support the assumption that the phylogenetic relations of the two new species are rather close, supported by (1) the inner element of the male P1 basis in both species is transformed into an elaborate hook-like structure; (2) the mandible basis of both species with a similar group of filaments; and (3) the maxilla with two endites and a proximal endite with a group of filaments.

Both new species appear to coexist next to each other, being collected from a reservoir at the mouth of a karstic spring. Their differentiation of morphological characters is slight, suggesting the close phylogenetic relatedness of both species (Karanovic and Cooper 2012; Sanoamuang et al. 2019). However, both species can represent “sink populations” when specimens would/could be drifted into the spring from different original habitats within the same catchment area (Culver et al. 2012).

The low degree of differentiation of morphological characters, although both new species belong to different morpho-groups (*chappuisi* vs. *vasconica* group), can be explained by occupation of different habitats (allopatric theory) within the same aquifer (sympatric theory), suggesting the relatively recent divergence of both species (Karanovic and Cooper 2012; Watiroyram et al. 2012). It is difficult to determine what the exact cause of the divergence is because the coexistence of most related species can occur in other ways, either as an allopatric (Karanovic and Cooper 2012) or a parapatric model of speciation (Gillespie 2004).

Once the sexual dimorphism is developed in appendages related to reproductive behavior/barrier (i.e., grasping mechanisms) in late copepodid or adult stages (Gilbert and Williamson 1983), the shape and configuration of the transformed element of the male P1 basis in the new species seem likely to be significant in terms of the taxonomic point of view, too. Probably, the modification has evolved and is used as a mating criterion to assess the potential mates, like species identity, development stage, sex, or even mechanosensory stimuli, to reduce the chances for interspecific mating.

Specific morphological structures can play an important role in determining reproductive success, shaping population genetic structure, and maintaining species boundaries (Tung et al. 2012). Copepods have very different reproduction structures between orders or even families. However, the process can be broken down into similar steps, such as the initial phase, the stimulation/courtship phase, the copula phase, and the postcopulatory mate guarding phase (Königshoff and Glatzel 2008). A study on *Parastenocarisphyllura* Kiefer, 1938, has shown that the female caudal rami are grasped by the male antennules in the initial and stimulation/courtship phases of the mating behavior (Glatzel and Schminke 1996), suggesting the significance of both structures in specific recognition. The morphological differentiation of the shape of the female caudal rami is an important clue, indicating the reproductive niche partitioning of the closely related species (Karanovic and Cooper 2012). However, sexual dimorphism on caudal rami associated with elaborated clasping mechanisms on males’ antennules is rather rare, or at least not yet recorded, in the family Ameiridae, while it is common in the family Canthocamptidae, for example, the genera *Attheyella* Brady, 1880; *Maraenobiotus* Mrázek, 1893; *Bryocamptus* Chappuis, 1929; and *Moraria* T. & A. Scott, 1893 (Boxshall and Evstigneeva 1994, Brancelj and Karanovic 2015, Novikov et al. 2023, Alekseeva and Timoshkin 2024).

In the case of the two new species, the expansion of the caudal rami in the female and the modification of the proximalmost seta of segment 5 of the A1 into a hook-like element on the male antennule of *N.grandicaudis* sp. nov. is highly characteristic, probably adapted for both the mechanosensory perception in mate recognition, which reduces the chances of interspecific mating (Buskey 1998), and for the firm grasping of the male that reduces the chances of female escape that has been reported in some other studied copepods both in Calanoida (e.g., *Centropagestypicus* Krøyer, 1849) and Cyclopoida (e.g., *Oithonadavisae* Ferrari F.D. & Orsi, 1984) (Titelman et al. 2007). From an evolutionary point of view, the expansion of the caudal rami of *N.grandicaudis* sp. nov. is very specific among the representatives of the genus, as the only significant difference in the shape of the caudal ramus in *Nitocrella* has primarily resulted from the shortening of the structure, not from the expansion in the vertical axis. However, the observed unique shape of the inner element on the P1 basis in both new species as well as in other species of the family Ameiridae (Janetzky et al. 1996; Boxshall and Halsey 2004) is not yet well understood in the mating process.

Lastly, huge differences have been seen in the male antennule of closely related species that live in similar but ecologically different areas. These differences have been observed in the genus *Pseudograeteriella* Sanoamuang, Boonyanusith & Brancelj, 2019, from Thien Duong Cave in central Vietnam (Sanoamuang et al. 2019). The male of *P.longiaesthetascus* Sanoamuang, Boonyanusith & Brancelj, 2019 bears much longer aesthetascs on the antennule when compared to the length of those of *P.longifurcata* (Tran & Chang, 2013) (Sanoamuang et al. 2019). The reduction in setation has been found in the caudal rami of the former, but not in the latter. The reduction of the armament has generally been encountered in subterranean copepods, and the elongation of the chemical sensing organ, like the aesthetascs on antennae, has widely been documented in troglo/stygobiotic arthropod taxa, like crayfish (Ziemba et al. 2003), cave crickets (Lavoie et al. 2007), and other insects (Gunn 2004). An elongation of the aesthetasc in *P.longiaesthetascus* is probably an evolutionary adaptation for sensing the intraspecific pheromone, secondarily evolved for living in subterranean environments.

## Supplementary Material

XML Treatment for Nitocrella grandicaudis

XML Treatment for Nitocrella thailandensis

## ﻿Additional information

### Author ORCIDs

Chaichat Boonyanusith https://orcid.org/0000-0003-1487-2160

Anton Brancelj https://orcid.org/0000-0002-8767-3894

Laorsri Sanoamuang https://orcid.org/0000-0003-0377-1225

### Data availability

All of the data that support the findings of this study are available in the main text.
